# Integrated Bioinformatics Analysis Identifies Robust Biomarkers and Its Correlation With Immune Microenvironment in Nonalcoholic Fatty Liver Disease

**DOI:** 10.3389/fgene.2022.942153

**Published:** 2022-07-14

**Authors:** Feng Zhang, Zhengwei Zhang, Yapeng Li, Yi Sun, Xinliang Zhou, Xiaoning Chen, Shibo Sun

**Affiliations:** Department of General Surgery, Second Affiliated Hospital of Harbin Medical University, Harbin, China

**Keywords:** non-alcoholic fatty liver disease, integrated bioinformatics analysis, immune microenvironment, differentially expressed genes, robust biomarkers

## Abstract

**Objective:** Nonalcoholic fatty liver disease (NAFLD) is a serious threat to human health worldwide. In this study, the aim is to analyze diagnosis biomarkers in NAFLD and its relationship with the immune microenvironment based on bioinformatics analysis.

**Methods:** We downloaded microarray datasets (GSE48452 and GSE63067) from the Gene Expression Omnibus (GEO) database for screening differentially expressed genes (DEGs). The hub genes were screened by a series of machine learning analyses, such as support vector machine (SVM), least absolute shrinkage and selection operator (LASSO), and weighted gene co-expression network analysis (WGCNA). It is worth mentioning that we used the gene enrichment analysis to explore the driver pathways of NAFLD occurrence. Subsequently, the aforementioned genes were validated by external datasets (GSE66676). Moreover, the CIBERSORT algorithm was used to estimate the proportion of different types of immune cells. Finally, the Spearman analysis was used to verify the relationship between hub genes and immune cells.

**Results:** Hub genes (CAMK1D, CENPV, and TRHDE) were identified. In addition, we found that the pathogenesis of NAFLD is mainly related to nutrient metabolism and the immune system. In correlation analysis, CENPV expression had a strong negative correlation with resting memory CD4 T cells, and TRHDE expression had a strong positive correlation with naive B cells.

**Conclusion:** CAMK1D, CENPV, and TRHDE play regulatory roles in NAFLD. In particular, CENPV and TRHDE may regulate the immune microenvironment by mediating resting memory CD4 T cells and naive B cells, respectively, and thus influence disease progression.

## Introduction

Nonalcoholic Fatty Liver Disease (NAFLD) is defined by the presence of hepatic steatosis in the absence of significant alcohol consumption or causes other than the metabolic disorders constituting the metabolic syndrome, which is a leading cause of chronic liver disease and affects about 10% of the world population (Sven et al., 2020). Hepatic pathologies of NAFLD range from simple hepatic steatosis to nonalcoholic steatohepatitis (NASH), even developing into liver fibrosis, liver cirrhosis, and hepatic carcinoma (Kabbany et al., 2017). With the increasing incidence of obesity, diabetes, hyperlipidemia, and cardiovascular disease, NAFLD has become increasingly prevalent, which represents the hepatic manifestation of metabolic syndrome. The global prevalence of NAFLD will be increasing soon. Despite the enormous burden on healthcare costs, there is no effective cure approved for NAFLD. Lifestyle interventions are recommended as first‐line management in guidelines, but it is difficult to achieve favorable and persistent outcomes in the real world regrettably (Polyzos et al., 2019).

With the drastic development of generation sequencing technologies, systems biology techniques including genomics, metabolomics, transcriptomics, and proteomics provide new insight into solving this task. An increasing number of studies have indicated that NAFLD is linked to metabolic disorders (Huang and Kong, 2021; Luukkonen et al., 2021; Osborne et al., 2021). Immunity is also involved in the development and progression of NAFLD (Barrow et al., 2021; Huby and Gautier, 2021; Song et al., 2021). Nonetheless, there is still a lack of insensitive and targeted biomarkers that may be widely used in the clinical setting, which causes significant challenges for clinical diagnosis and treatment, especially for early diagnosis and follow-up strategy.

Therefore, an exploration into the molecular mechanism in NAFLD is necessary. To address these issues, we applied the GEO database to mine DEGs between NAFLD patients and normal patients, and determined the correlation between robust biomarkers, immune microenvironment and nutrient metabolism. Using various bioinformatics analysis methods, we described the differential genes and verified these genes in the external gene dataset, and finally screened CAMK1D, CENPV, and TRHDE. In addition, we found compounds or environmental poisons that might have a potential relationship with hub genes in the comparative toxicology database, which provided an important theoretical basis for the primary prevention and treatment of NAFLD.

## Materials and Methods

### Datasets and Data Preprocessing

Raw transcriptomic data from two microarray datasets (GSE48452 and GSE63067) based on the GPL11532 and GPL570 platforms, both taken from the liver tissue, were downloaded from the GEO database. Normalization was performed on the raw data using the sva package. PCA showed that the aforementioned analysis method was better at eliminating batch effects (Supplementary Figure.S1). Twenty-one healthy liver control tissues, as well as 27 NAFLD liver tissue samples, were ultimately included in the screening set. In addition, we downloaded the GPL6244 platform–based GSE66676, derived from liver wedge biopsies, as an external validation dataset. Particular clinical characteristics of the patients in the dataset are presented in Supplementary Datasheet S1.

### Screening and Validation of Hub Markers

As previous studies have done (Hu et al., 2022; Jiang et al., 2022), differentially expressed genes (DEGs) were screened in GSE48452 and GSE63067 in a batch-calibrated screening set. DEGs between NAFLD samples and normal samples were screened using the limma program package, with P. adj. value <0.05 selected as the cutoff criterion. Considering the situation of datasets, we did not set logFC as the threshold. Subsequently, the core genes were further screened in the aforementioned DEGs using a 10-fold cross-validation of LASSO (glmnet package). Alternatively, support vector machine–recursive feature elimination (SVM-REF) is a support vector machine–based machine learning method that builds on DEGs by removing support vector machine–generated feature vectors (e1071 and msvmRFE program packages) to find the optimal core genes. Simultaneously, we screened DEGs using one-way logistic regression with NAFLD as the dependent variable, using *p* < 0.001 as the threshold. In the WGCNA analysis, all DEGs satisfying *p*. value <0.05 in normal and NAFLD samples were used as input, and each sample clustered well, with a shear line of 30 as the threshold, and one outlier sample was excluded. Subsequently, a soft threshold from 1 to 20 was used for topology calculation to determine the optimal soft threshold of 6. The curve is smoothest when β was 6. Based on the soft threshold, the relationship matrix was converted to an adjacency matrix and then to a topological overlap matrix (TOM) for mean linkage hierarchical clustering, and the related modules were classified according to TOM with the number of genes in each module not less than 50. The gene module shear height in this study was 0.7, and similar module merging was performed. In addition, GS and GS of each module were calculated. In addition, GS and MM within each module were calculated for scatter plots. Finally, the Pearson method was used to calculate the correlation between the merged modules and the occurrence of NAFLD.

### Enrichment Analysis

GO enrichment analysis is a common bioinformatics method used to search for comprehensive information on large-scale genetic data, including BP, CC, and MF. In addition, KEGG pathway enrichment analysis is widely used to understand biological mechanisms and functions. At the same time, DO enrichment analysis can be used to explore the diseases in which the genes of interest are predominantly involved. Finally, GO, KEGG pathway, and DO analyses were visualized using the GO plot program package. Finally, primary signaling pathways associated with core genes were further explored using the cluster profile package and the GSVA package. The h.all.v7.4.symbols.gmt gene set was downloaded from MSigDB, and the gene set was subjected to GSVA analysis with the gene expression matrix to explore the regulatory pathways that may be involved.

### Construction of Hub Gene Regulatory Network

First, potential miRNAs targeting hub genes were predicted using mirDIP and Starbase databases, with the threshold set to minimum score = very high, to identify the regulatory network of miRs downstream of core genes. In addition, the TRRUST database contains 800 human transcription factors (TFs), and TF-core gene reciprocal pairs with *p*-values < 0.05 were selected to build upstream regulatory networks. In addition, we queried the Comparative Toxicogenomic database for compounds or environmental reads that might retain potential relationships with core genes. Finally, the core gene regulatory network was visualized based on the Networkanalyst database (Supplementary Figures S2, S3).

### Immune Analysis Algorithm

As previous studies have done (Lu et al., 2021; Shen et al., 2021), the CIBERSORT algorithm calculates the proportion of different immune cell types based on the expression levels of immune cell–related genes. The output of the 22 infiltrated immune cells was integrated to generate a matrix of immune cell fractions for analysis (the CIBERSORT program package). The correlation of core genes with the content of the 22 immune cell types was calculated using the Spearman method.

### Statistical Analysis

All statistical analyses were performed using R software (v.4.0.1). Detailed statistical methods for transcriptome data processing are covered in the aforementioned section. *p* < 0.05 was considered statistically significant.

## Result

### DEGs in Different Datasets

**FIGURE 1:**
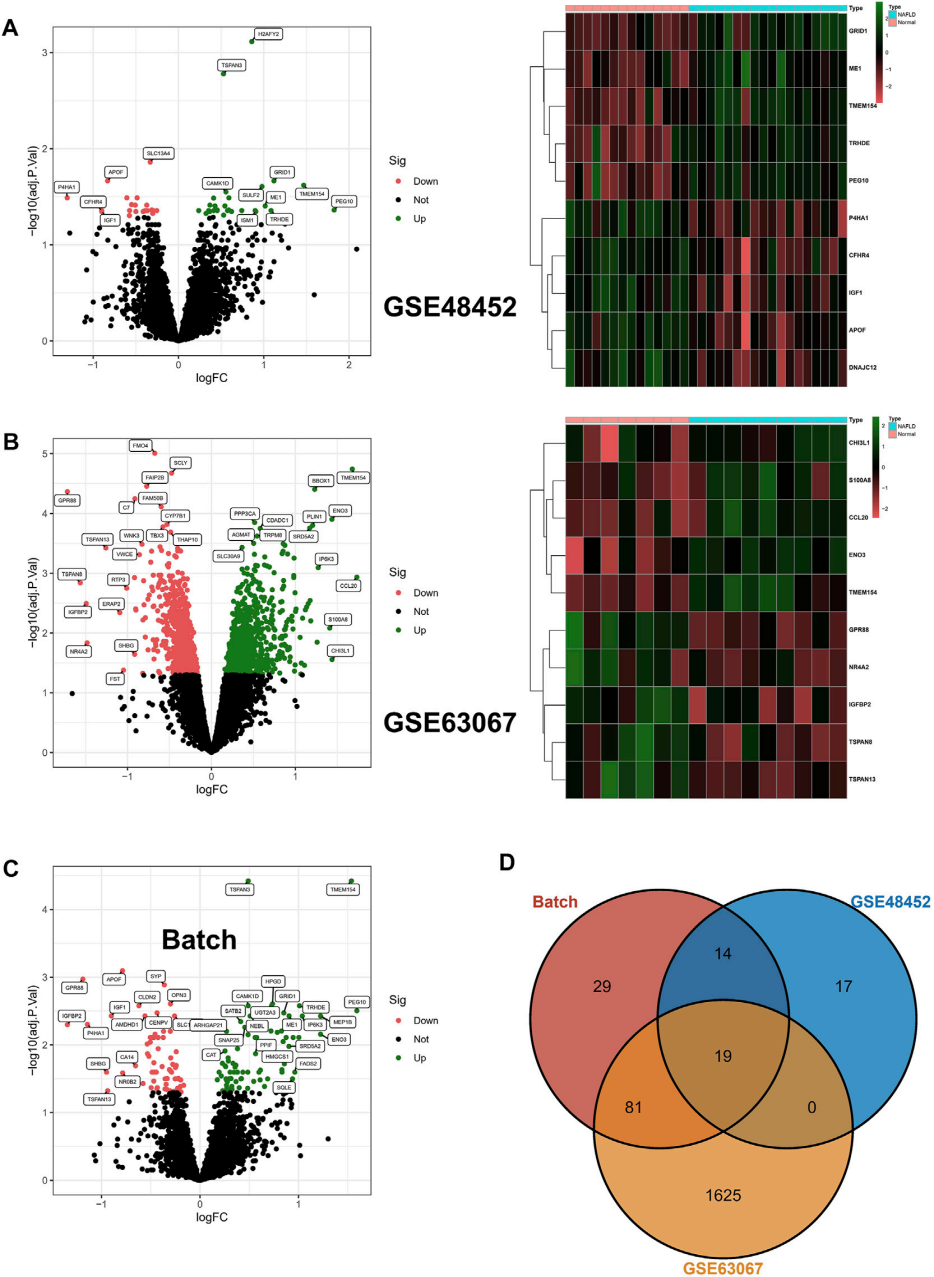
Differentially expressed genes. **(A,B)** Differentially expressed genes (DEGs) were identified in GSE48452 and GSE63067, respectively, with upregulated genes indicated in red and downregulated genes in green in the volcano plot; in addition, the heat map shows the top 10 differentially expressed genes. **(C)** After batch correction, we again identified DEGs in the screening set using the limma package, and the volcano plot in red indicates upregulated genes and green indicates downregulated genes. **(D)** The DEGs from the three aforementioned screens were crossed, resulting in the identification of 19 core DEGs.

In the beginning, we identified 50 differentially expressed genes (DEGs) in GSE48452, and the volcano map shows 28 upregulated genes as well as 22 downregulated genes; in addition, the heat map shows the top 10 differentially expressed genes (Figure 1A). In addition, 1725 DEGs were identified in GSE63067, and the volcano map and heat map demonstrates 885 upregulated genes as well as 840 downregulated genes (Figure 1B). In addition, after batch correction, we again identified DEGs in the screening set using the limma package, with the volcano map demonstrating 77 upregulated genes as well as 66 downregulated genes (Figure 1C). Ultimately, we crossed DEGs from the three aforementioned screens and ultimately identified 19 core DEGs. In detail, TMEM154, TSPAN3, CAMK1D, TRHDE, PEG10, ME1, SATB2, SNAP25, ANKRD18A, ISM1, and SGCB were upregulated in NAFLD, while APOF, SYP, OPN3, CENPV, IGF1, AMDHD1, P4HA1, and MRPL21 were downregulated within NAFLD samples (Figure 1D).

### Enrichment Analysis in DEGs

To explore the potential biological mechanisms of the 19 DEGs and the development of NAFLD, KEGG analysis illustrated the possible biological mechanisms of NAFLD development such as glioma, hypertrophic cardiomyopathy, and other disease processes (Supplementary Figure S2A). In addition, DO analysis revealed 19 differential genes that may have shared pathogenesis in diseases such as cell type benign neoplasm (Supplementary Figure S2B). Meanwhile, the BP section of the GO enrichment analysis suggested the important role of the dicarboxylic acid metabolic process, pyruvate metabolic process, etc. (Supplementary Figure S2C). Finally, we downloaded the corresponding gene sets from MSigDB and performed the GSVA analysis of the gene sets and gene expression matrices to explore the potential pathways involved in the pathogenesis of NAFLD, and the results showed that allograft rejection, cholesterol homeostasis, complement, and inflammatory response pathways have significant roles (Supplementary Figure S2D). Interestingly, taken together, the established chain of evidence suggests a possible involvement of the immune system with the nutritional metabolic system in NAFLD.

### Integrated LASSO Analysis, Machine Learning Algorithm, and Logistic Analysis for Screening Hub Biomarkers

**FIGURE 2:**
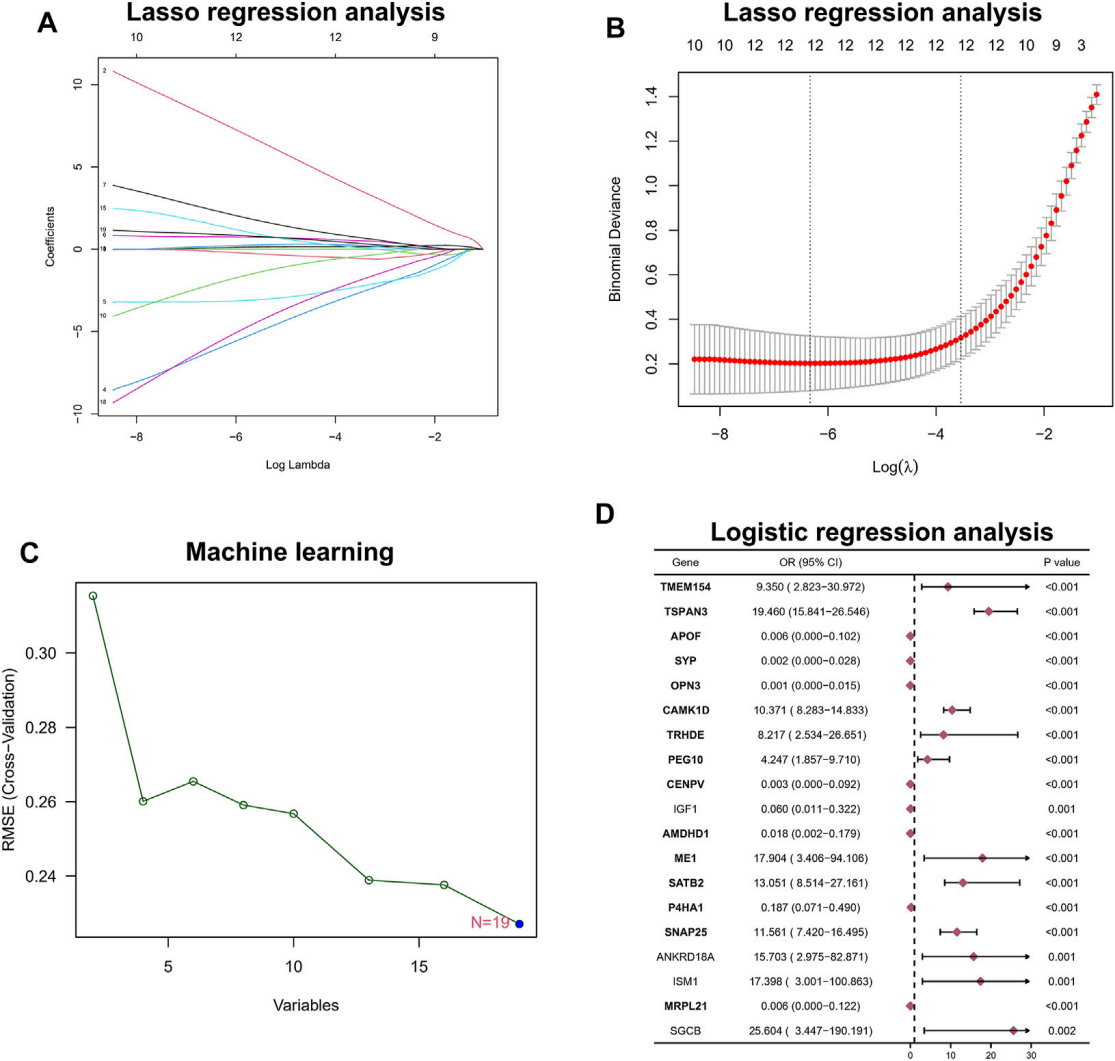
Hub biomarker screening. **(A,B)** LASSO regression analysis. **(C)** Machine learning approach with SVM. **(D)** Logistic regression analysis.

Among the aforementioned 19 DEGs, we further screened the core genes using a 10-fold cross-validation of LASSO and finally screened 12 potential genes (Figures 2A,B). At the same time, we performed an in-depth screening of the differential genes using a machine learning approach with SVM, and the results showed the lowest RMSE values when all 19 genes were included (Figure 2C). Finally, we performed a one-way logistic analysis of the expression of the 19 DEGs, with NAFLD occurrence as the dependent variable, and the final results of Moritu showed that 15 genes entered the subsequent analysis (Figure 2D).

### WCGNA Analysis Was Used for Further Screening

**FIGURE 3:**
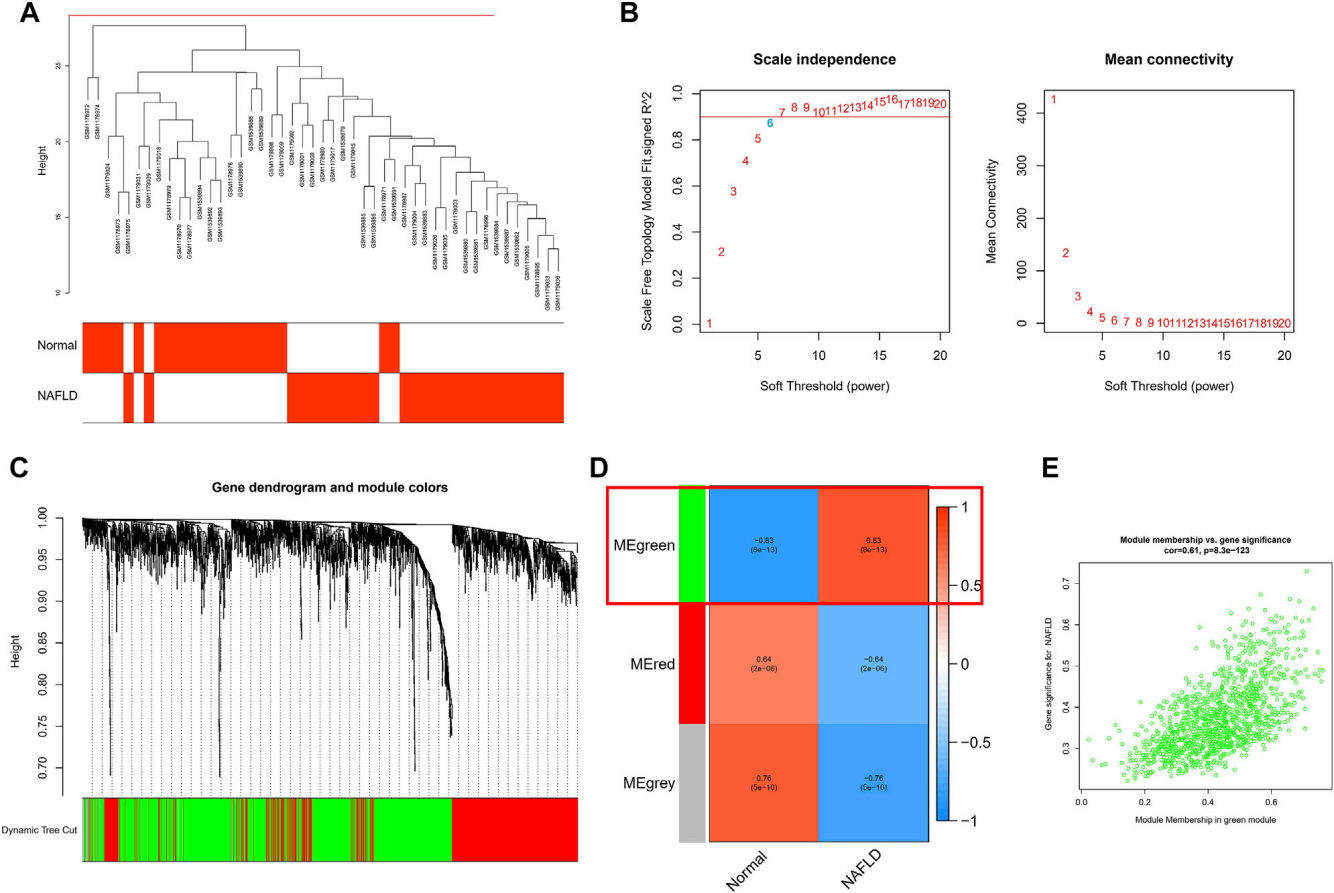
WCGNA analysis was used for further screening. **(A)** Each sample clustered well, with a shear line of 30 as the threshold and one case of outlier removed. **(B)** Topological calculations with soft thresholds from 1 to 20 to determine the optimal soft threshold of 6. **(C)** Based on soft thresholds, the relationship matrix is converted to an adjacency matrix and then to a topological overlap matrix (TOM) for average link hierarchy clustering, which classifies the relevant modules according to TOM. **(D)** Calculating the correlation between genes within modules and clinical traits, we found that the green module had the highest correlation with the occurrence of NAFLD (*p* = 0.83). **(E)** The scatter plot of the correlation between GS and MM for 1,196 genes within the green module.

To further link clinical information to key genes, the expression of only 1,989 genes that met *p*. value <0.05 in the analysis of differences between normal and NAFLD samples was used as the input matrix in WGCNA analysis. The samples clustered well, and one outlier sample was excluded using a shear line of 30 as the threshold (Figure 3A). Subsequently, a soft threshold of 1–20 was used for topological calculations, and the optimal soft threshold was determined to be 6 (Figure 3B). Based on the soft threshold, the relationship matrix was converted to an adjacency matrix and then to a topological overlap matrix (TOM) for mean linkage hierarchical clustering, and the related modules were classified according to TOM with no less than 50 genes in each module, and the similar gene modules were finally merged (Figure 3C), resulting in the identification of three modules. In addition, to calculate the correlation between genes within modules and clinical traits, we found that the green module included the highest correlation with the occurrence of NAFLD (*p* = 0.83), so this was used as the core module (Figure 3D). In addition, GS and MM were calculated for 1,196 genes within the green module, and correlation scatter plots were drawn (Figure 3E). We found a direct correlation between GS and MM of genes within the core module, which verified our speculation from another perspective.

### Exploring Predictive Value of Biomarkers

**FIGURE 4:**
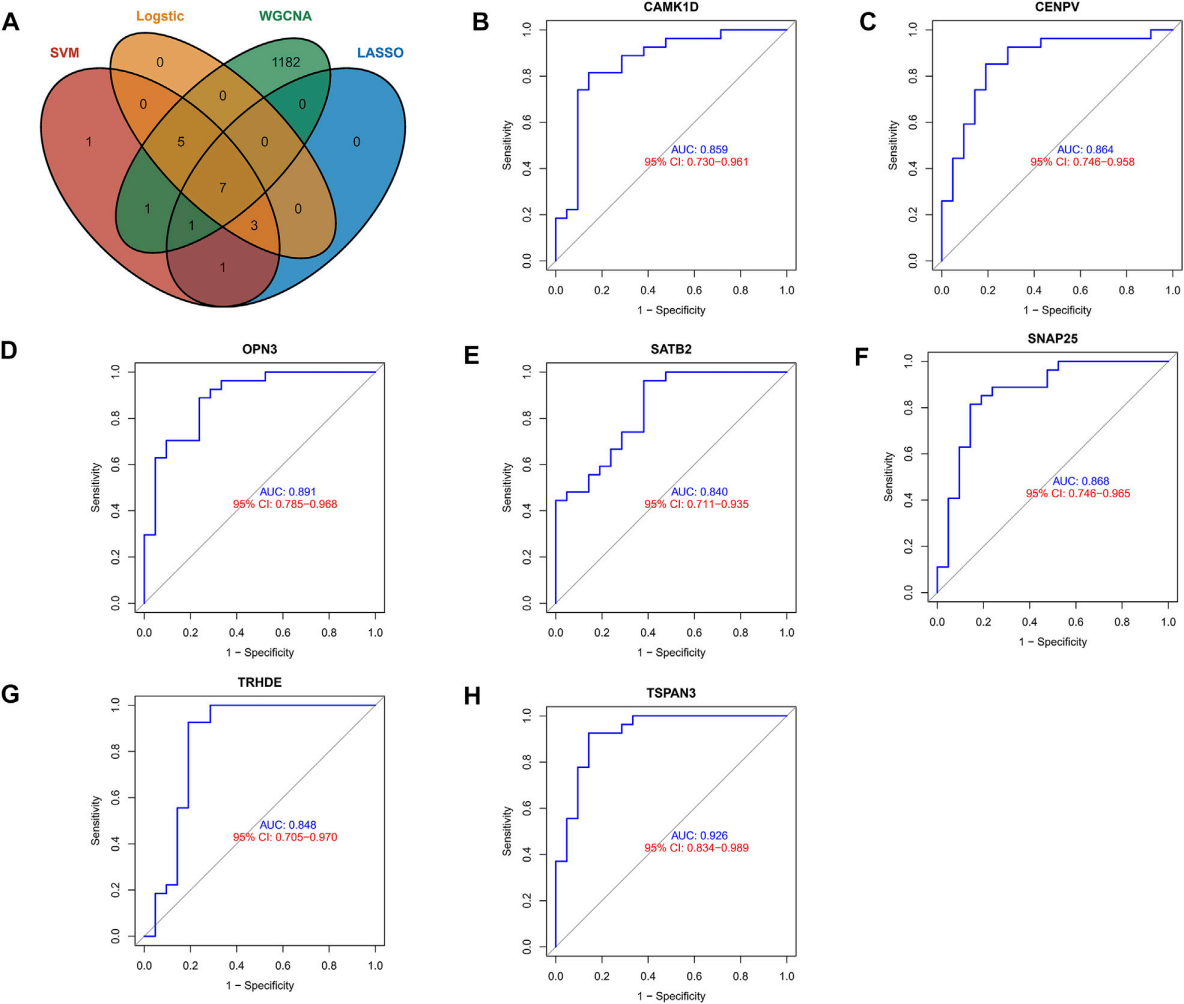
Exploring the predictive value of biomarkers. **(A)** Cross-tabulation of relevant genes from WGCNA, LASSO, Logistic, and machine learning to identify candidate genes. **(B–H)** ROC analysis of the aforementioned genes.

To identify core biomarkers, we cross-tabulated relevant genes from WGCNA, LASSO, Logistic, and machine learning, and finally identified seven biomarkers as our candidate genes (Figure 4A). We performed ROC analysis on each of these seven genes in the screening set, and the results showed that all genes had excellent predictive performance in the screening set: CAMK1D (AUC = 0.859, Figure 4B), CENPV (AUC = 0.864, Figure 4C), OPN3 (AUC = 0.891, Figure 4D), SATB2 (AUC = 0.840 (Figure 4E), SNAP25 (AUC = 0.868, Figure 4F), TRHED (AUC = 0.848, Figure 4G), and TSPAN3 (AUC = 0.926, Figure 4H).

### The Validation of Hub Biomarkers

**FIGURE 5:**
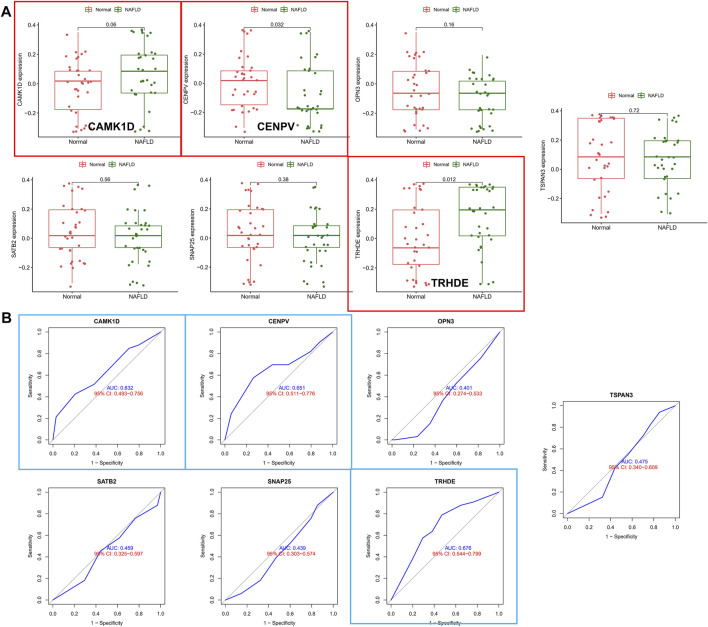
Validation of hub biomarkers. **(A)** Candidate genes were validated in the external validation set, and those marked in red indicate differential expression between samples. **(B)** ROC analysis of candidate genes in the external dataset, with blue annotations indicating good predictive performance for the gene.

To validate the accuracy of seven aforementioned genes, we performed validation in an external validation set. In the dataset, also with liver tissue sequencing, only CAMK1D, CENPV, and TRHDE obtained differential expression between samples (Figure 5A), and in addition, as shown in Figure 5B, ROC analysis also demonstrated better predictive performance for three aforementioned biomarkers (CAMK1D, AUC = 0.632; CENPV, ADU = 0.651; TRHED, ACU = 0.676). In addition, we queried the Comparative Toxicogenomics database for compounds or environmental toxicants that may have potential relationships with core genes. Finally, the core gene regulatory network was visualized based on the Networkanalyst database (Supplementary Figures S3, S4).

### The Analysis of Differences in Immune Microenvironment

**FIGURE 6:**
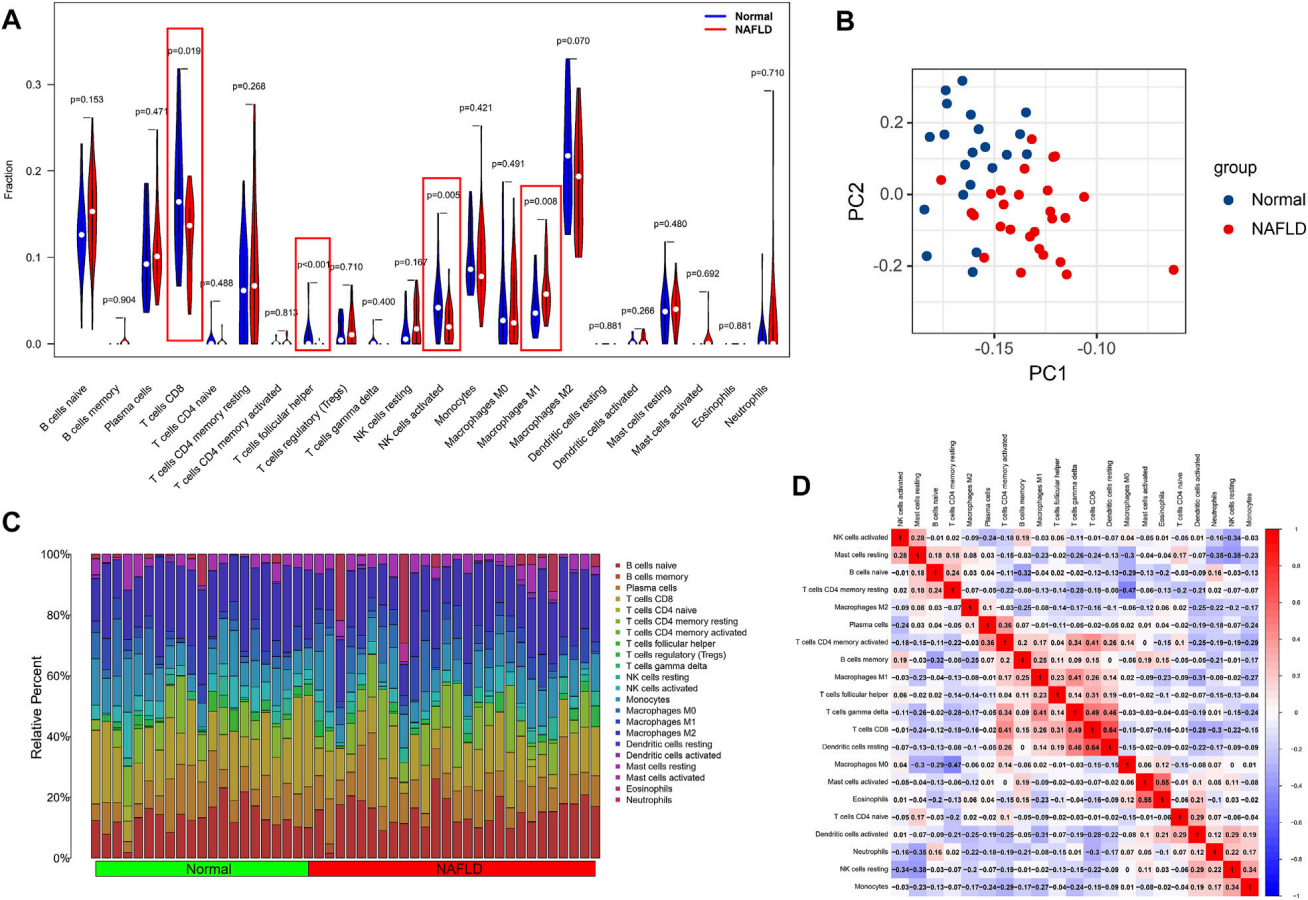
Analysis of differences in the immune microenvironment. **(A)** The CIBERSORT algorithm was used to analyze the content of immune cells in different tissues, and the red markers are the immune cells with different content in the two different samples. **(B)** PCA analysis of the distribution of different immune cells in NAFLD and normal tissue samples. **(C)** A holistic view of the distribution of immune cells between different tissues. **(D)** Correlation analysis was performed on all immune cells in the CIBERSORT algorithm.

Considering the important role of the immune pathway in NAFLD in the GSEA gene enrichment analysis, we used the CIBERSORT algorithm to analyze the immune cell content in various tissues. The results indicated higher levels of CD8 T cells, activated NK cells, and follicular-helper T cells in normal samples; in NAFLD tissues, only Macrophages M1 had a higher enrichment fraction compared to normal liver tissue (Figure 6A). In addition, the results of PCA analysis also showed a natural heterogeneity in the distribution of immune cells between the two tissues (Figure 6B). At the same time, the bar chart illustrates the general landscape of immune cell distribution between the different tissues (Figure 6C). Finally, as shown in Figure 6D, we performed a correlation analysis of all immune cells in the CIBERSORT algorithm, showing Macrophages M0 had the strongest negative correlation with T cells CD4 memory resting (*r* = −0.47) and T cells CD8 had the strongest positive correlation with Dendritic cells resting (*r* = 0.64).

### Correlation Hub Biomarkers With Immune Infiltrating Cells

**FIGURE 7:**
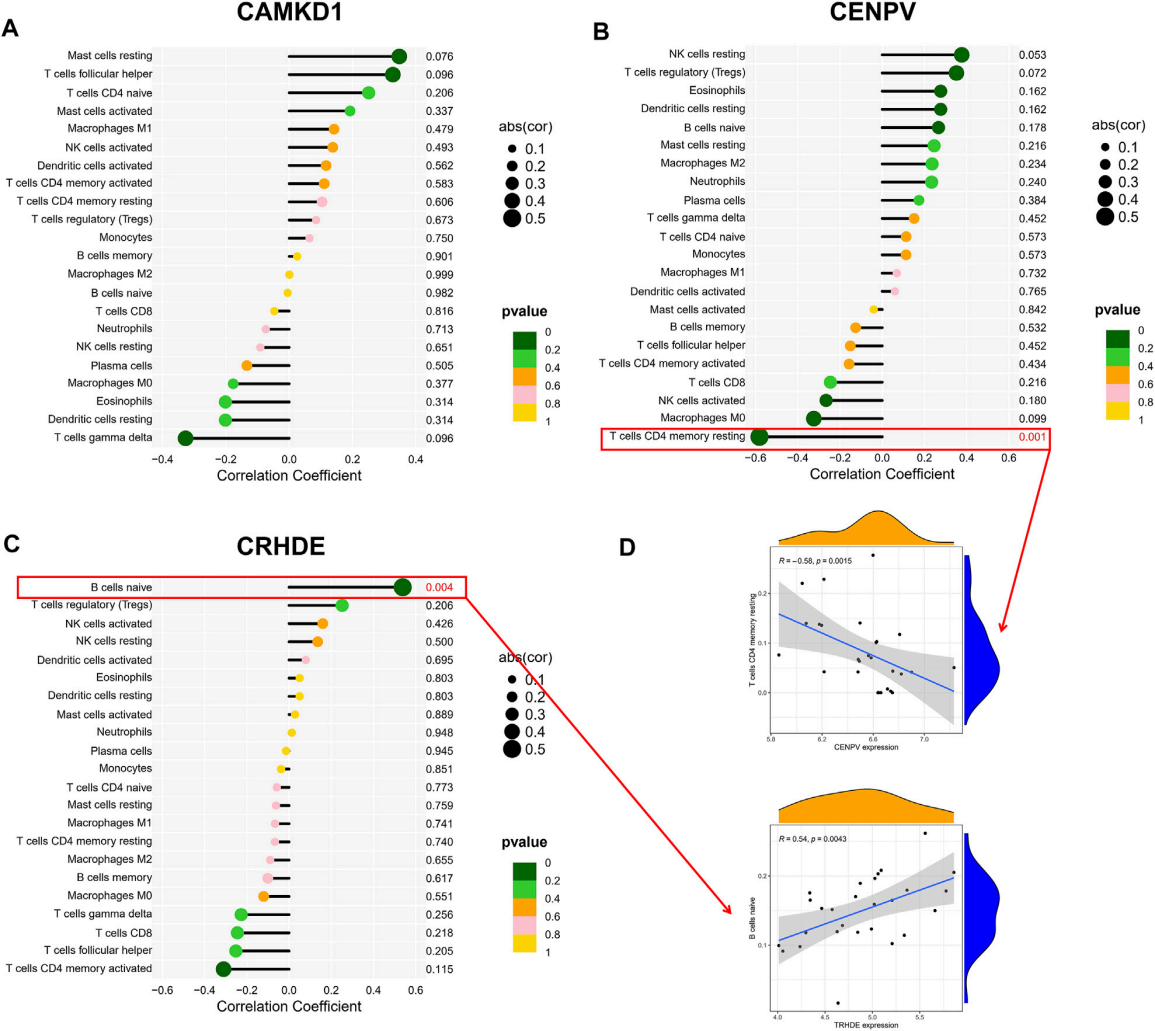
Correlation hub biomarkers with immune-infiltrating cells. **(A–C)** Analysis of the correlation between the hub gene and immune cell content, with those marked in red indicating a statistically significant correlation between the gene and immune cells. **(D)** Once again, statistically significant correlations were made between hub genes and immune cell content.

To explore the association of our identified core genes CAMK1D, CENPV, and TRHDE with immune cell content, we performed separate correlation analyses. Unfortunately, there was no statistically significant correlation between the CAMKD1 expression and the content of 22 types of aforementioned immune cells (Figure 7A). In addition, the CENPV expression had a significant negative correlation with resting memory CD4 T cells, *r* = −0.581 (Figures 7B,D). Simultaneously, TRHDE expression had a strong positive correlation with naive B cells, *r* = 0.538 (Figures 7C,D). Based on our results, we propose a speculation that the core genes CENPV and CRHDE may be involved in disease progression and regulate the immune microenvironment by mediating resting memory CD4 T cells and naive B cells, respectively.

## Discussion

NAFLD is a disease spectrum of a series of liver diseases, including simple fatty infiltration (steatosis) and fat and inflammation [nonalcoholic steatohepatitis (NASH)), and cirrhosis] without excessive alcohol consumption (<20 g a day for women and <30 g a day for men is adopted). NAFLD is associated with metabolic syndrome, including insulin resistance, hyperlipidemia, type 2 diabetes, and obesity. It is considered to represent the hepatic manifestation of this syndrome (de Alwis and Day, 2008; Anstee et al., 2011). An epidemiological model predicts that the prevalence of NAFLD/NASH will continue to increase and the mortality of associated diseases will double by 2030 (Estes et al., 2018). NAFLD is gradually becoming the fastest-growing cause of HCC; many risk factors for NAFLD are also independently associated with HCC, and screening for NAFLD-related HCC is difficult, so the exploration of the pathogenesis of NAFLD, related biomarkers, primary treatment, and prevention is urgent (Ioannou, 2021). In our study, we applied the GEO database to mine differential genes in NAFLD patients versus normal patients to identify strong biomarkers for correlation with the immune microenvironment. We characterized the differential genes in NAFLD patients versus normal patients by using various raw letter analysis methods and validated these differential genes in an external genetic dataset, finally screening for CAMK1D, CENPV, and TRHDE, and illustrating the relevance of these biomarkers to the immune microenvironment. In addition, we queried the Comparative Toxicogenomics database for compounds or environmental toxicants that may have potential relationships with core genes, providing a historic theoretical basis for the primary prevention and treatment of NAFLD.

Previous studies have shown that among multiple genetic risk factors, an SNP in the gene-encoding patatin-like phospholipase domain-containing 3 (PNPLA3) strongly predicts an increased risk of developing NAFLD. The G allele of the PNPLA3 rs738409 (148M) variant is associated with an increased risk of NAFLD development, and progression of NAFLD to NASH, liver fibrosis, and even cirrhosis (Romeo et al., 2008; Valenti et al., 2010). Epidemiological studies have shown that fatty acids (FAs) and palmitoleic acid levels in NAFLD patients predict increased risk factors for CVD-related mortality and that the principal driver of CVD in NAFLD patients is a mutagenic lipid profile caused by increased hepatic lipogenesis. However, the specific pathogenesis of dilated cardiomyopathy and hypertrophic cardiomyopathy associated with the NAFLD process is unknown and needs to be further explored (Lai et al., 2019; Soehnlein and Libby, 2021). On the other hand, plasma amino acid concentrations have been associated with the pathogenesis of NAFLD and the progression of NASH, but the exact mechanisms are unclear. Concentrations of AA are altered in metabolic diseases such as T2DM, NAFLD, and obesity, and an established chain of evidence suggests that AA concentrations are associated with insulin resistance. BCAA has been of interest and may play a role in promoting peripheral and hepatic insulin resistance and in accelerating the T2DM process. In obese patients with NAFLD, fasting BCAA levels are elevated and associated with peripheral insulin resistance, possibly in relation to the liver being the site of protein and amino acid metabolism. In contrast, however, serine and glycine are found to be reduced in metabolic diseases such as NAFLD, suggesting that glycine metabolism is associated with the pathogenesis of NAFLD (Hyötyläinen et al., 2016; Gaggini et al., 2018). In our study, through the KEGG analysis of NAFLD, the DO analysis of the screened differential genes, GO analysis, and GSVA analysis, we found that the pathogenesis of NAFLD may involve the immune system and the nutritional metabolic system.

To further validate the correlation between DEGs screened from the database and NAFLD, LASSO regression analysis, machine learning, and logistic regression analysis were performed on 19 DEGs. Machine learning with SVM was chosen over other tools because of its ability to function with extraordinary accuracy and effective model. In addition, SVM machine learning has a nonlinear processing characteristic that allows it to produce a more accurate output than other algorithms, even when the data contain a large variability. However, in this study, when using the SVM’s machine learning approach for in-depth screening of DEGs, RMSE only showed a minimum when all genes were included. The weighted gene coexpression network analysis (WGCNA) is a simple method that allows the construction of gene expression networks by aggregating highly related genes into modules, a method that allows visualization of the most representative AMI genes. These core elements of the biological network are more likely to represent essential genes with more critical functions (Langfelder et al., 2011). In order to identify the core biomarkers, we crossed the relevant genes screened by the previous methods and identified a total of seven candidate genes, and after ROC analysis, we found that all seven genes had strong predictive power. ROC analysis also demonstrated good predictive power for these three core biomarkers. We have predicted potential candidate compounds for these three hub genes, which are important for both primary prevention and subsequent targeted therapy in patients.

The CAMK1 family of calmodulin-dependent kinases is widely expressed in hepatocytes, endothelial cells, immune cells, and the essential nervous system (CNS) (Parkinson et al., 2007; Wayman et al., 2008). CAMK1D may play a role in hepatic gluconeogenesis (Rausch et al., 2018). Lina Xu et al., using integrated Hi-C, Nanopore, and RNA sequencing techniques to analyze liver tissues from normal and NAFLD mice, found thousands of regions in the genome with 3D chromatin organization and genomic rearrangements in the genome and revealed genetic dysregulation accompanying these variants. These genes were identified in NAFLD and were affected by genetic rearrangements and spatial organization disruption. Among them, CAMK1 expression was downregulated by alternating CNV and SV, chromatin loop, domains, and interaction matrix (Xu et al., 2021). In the type 2 diabetes CDC123/CAMK1D GWAS (genome-wide association studies) locus, rs11257655 affects transcriptional activity by altering the binding of the protein complexes of FOXA1 and FOXA2, a potential molecular mechanism in type 2 diabetes (Fogarty et al., 2014). Christophe Fromont et al. first validated CAMK1D as a target for diabetes therapy in an *in vivo* experiment (Fromont et al., 2020). A single nucleotide polymorphism (SNP) genotyping of 11,530 cases pointed out that SNP rs10906115A of CDC123/CAMK1D was significantly associated with susceptibility to type 2 diabetes in the Japanese population (Imamura et al., 2011). However, the specific mechanism of regulation of NAFLD by CAMK1D is unclear. CENPV is a component of mitotic chromosomes associated with cytoplasmic microtubules. Elena Chiticariu et al. found that CENPV localizes to primary cilia in interphase, regulates cilia levels of acetylated microtubulin (α-tubulin), and is overexpressed in basal cell carcinomas and adnexal skin tumors (Chiticariu et al., 2020). CENPV levels are critical for cell viability, and either decreased or increased protein levels lead to cell death. cENPV provides an interesting link between the chromosomal passenger complex (CPC), primary contraction of mitotic chromosomes, and peristomal heterochromatin. The depletion of CENPV leads to a strong CPC phenotype (difficulties in chromosome bi-orientation and a failure to complete cytokinesis), followed rapidly by apoptotic cell death (Tadeu et al., 2008). The function of the CENPV gene is more organelle-specific and its role in the regulation of NAFLD has not yet been reported, and determining its detailed role and mechanism remains an exciting challenge for subsequent research. TRHDE was reported to be a DNA methylation marker for precancerous lesions in oral cancer (Shridhar et al., 2016). The overexpression of the noncoding long RNA TRHDE-AS1 inhibits lung cancer progression via the miRNA-103/KLF4 axis (Zhuan et al., 2019). In a study of the genomic signature of gliomas, TRHDE was found to be positively correlated with the disease pathogenesis process (Liang et al., 2017). In a study of the genetic basis of thyrotropin receptor antibodies and hyperthyroidism in mice immunized with CXB recombinant inbred strains, it was uncovered that the TRHDE expression is controlled by thyroid hormones and is linked to genes related to thyroid function, which represents an extremely intriguing result (Aliesky et al., 2006). There are no studies on the role of TRHDE in NAFLD, but it has been reported in oral cancer, lung cancer, and glioma development, and its relationship with thyroid hormones could be explored in depth.

Inflammation is a hallmark of NAFLD that continues to progress to NASH and is characterized by a severe dysregulation of different innate and adaptive immune cell compartments, with immune cells regrouping in the liver and being activated (Parthasarathy et al., 2020). We have presently obtained two alternative views on the inflammatory response in NAFLD. While dysregulated immune cells can further exacerbate liver damage, the inflammatory response that occurs early in the process of liver injury may be substantial for tissue healing and repair (Wynn and Vannella, 2016; Eming et al., 2017). Our analysis of the immune cell content of NAFLD and normal tissues showed that normal tissues had higher levels of T cells CD8, activated NK cells, and follicular-helper T cells, while only Macrophages M1 had a relatively high enrichment fraction in NAFLD samples. A study in triple-transgenic model pigs suggests that CD8 T cells play a crucial role in adipose inflammation, recruiting and activating macrophages after activation in adipose tissue, which differs from our results for several reasons; we speculate, first, that CD8 T cells may not be consistently highly expressed throughout the development of the disease. Second, CD8 T cells may act more early in the development of NAFLD, and the exact cause and mechanism may need to be further explored (Zhang et al., 2021). NK cells perform a fundamentally meaningful role in liver fibrosis and are generally thought to reduce fibrotic events by eliminating activated hepatic stellate cells or altering the phenotype of hepatic macrophages. However, previous studies have found NK cell dysfunction in some patients with hepatocellular carcinoma and an association with a poor prognosis (Cai et al., 2008). In an analysis of the differences in NK cell surface markers and cell function correlations between NAFLD and ordinary volunteers, it was discovered that peripheral blood NK cells from NAFLD patients had reduced abundance and function (Sakamoto et al., 2021). The regulatory role of follicular-helper T cells (Tfh) is more in viral and autoimmune hepatitis. Xiaowen Wang et al. showed that in studies of blood from HBV-infected mice and patients with chronic HBV infection, the Tfh cell response to HBsAg was required for HBV clearance and that this response was blocked. The inhibition of Treg cell activity with anti-CTLA4 neutralizing antibodies restored the ability of Tfh cells to acquire HBV infection and could be implemented in the treatment of chronic HBV-infected patients. The dysregulation of the immune response to Tfh also induces lethal autoimmune hepatitis (Wang et al., 2018). The role of pro-inflammatory Macrophage M1 in NASH is primarily to exert immunomodulatory activity, with Macrophage M1 accumulating in areas of inflammation to secrete pro-inflammatory factors that exacerbate the progression of inflammation (Sun et al., 2021). The increase or decrease in the number of these immune cells can only suggest the occurrence of immune dysregulation in NAFLD tissues and the recruitment of some immune cells, which can be useful for subsequent studies and can be used clinically to slow down the progression of NAFLD or reverse the disease process to some extent by regulating the level of immune cells. The most significant negative correlation was demonstrated between Macrophages M0 and resting memory CD4 T cells, indicating that Macrophages M0 may be related to the activation of resting memory CD4 T cells, CD8 T cells, and dendritic cells. The most direct positive correlation between CD8 T cells and dendritic cells resting suggests that CD8 T cells inhibit dendritic cell recruitment through immune cell interactions during the NAFLD recruitment. However, this is only speculation and further experiments are needed to verify the exact relationship and mechanism of action.

Is there a relationship between the infiltration of immune cells and the screened hub biomarkers? To address this question, we analyzed the correlation between CAMK1D, CENPV, and TRHDE and immune cells, and finally found that CENPV expression had a direct negative correlation with resting memory CD4 T cells, and TRHDE expression had a strong positive correlation with naive B cells; we venture to guess that CENPV and TRHDE may regulate resting memory CD4 T cells and naive B cells through certain pathways, and have an impact on disease progression remission or recovery in NAFLD in terms of immune and inflammatory responses. Despite our findings, our conclusions need to be adopted with caution due to the limitations of our study. Our study is limited to the processing of previous data, and the timeliness and accuracy of our findings need to be verified, which may provide a reference for clinical diagnosis, but more detailed basic experiments and clinical trials are needed to support our findings before they can be applied to clinical treatment. Moreover, NAFLD samples are difficult to obtain, and it is difficult for us to conduct more assays.

In summary, we screened the GEO database for differential genes in two datasets, GSE48452 and GSE63067, and performed LASSO regression analysis, SVM machine learning analysis, logistic regression analysis, and WGCNA analysis on the differentially expressed genes. Seven candidate genes (CAMK1D, CENPV, OPN3, SATB2, SNAP25, TRHED, and TSPAN3) were finally screened, and three hub genes (CAMK1D, TRHDE, and CENPV) were identified after the external dataset validation. In GO analysis, we found that the disease process in NAFLD is strongly associated with nutritional metabolism and the immune system, and we identified more excessive levels of macrophage M1 in NAFLD than in normal tissue through immune cell content analysis. The ultimate analysis of hub genes and immune cell correlations suggests that CENPV and TRHDE may influence the disease process in NAFLD by regulating resting memory CD4 T cells and naive B cells through certain pathways. This may additionally provide a theoretical basis for subsequent clinical treatment.

## Data Availability

The original contributions presented in the study are included in the article/Supplementary Material; further inquiries can be directed to the corresponding authors.
