# Supplementary figures and images for: Integrated Bioinformatics Analysis Identifies Robust Biomarkers and Its Correlation With Immune Microenvironment in Nonalcoholic Fatty Liver Disease
Source: Front Genet. 2022 Jul 14;13:942153. doi: 10.3389/fgene.2022.942153 (PMC9330026; doi:10.3389/fgene.2022.942153)

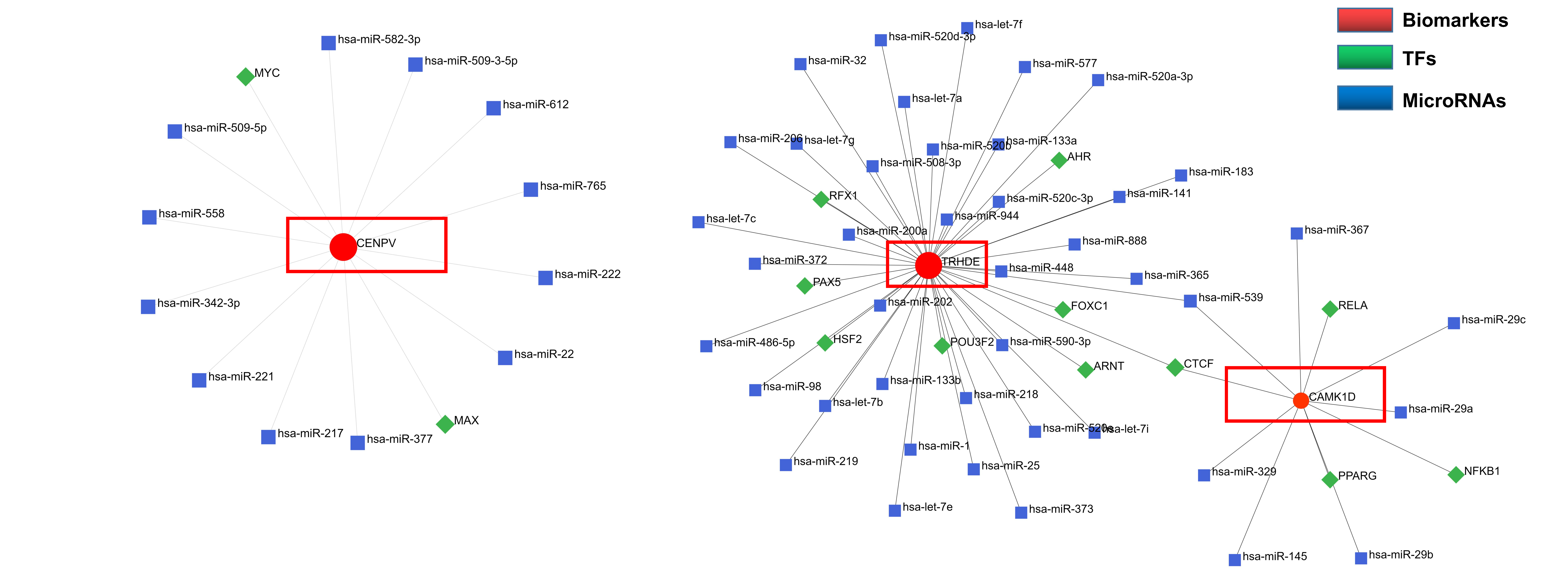

Supplement: Supplementary file 1 [file Image3.TIFF]

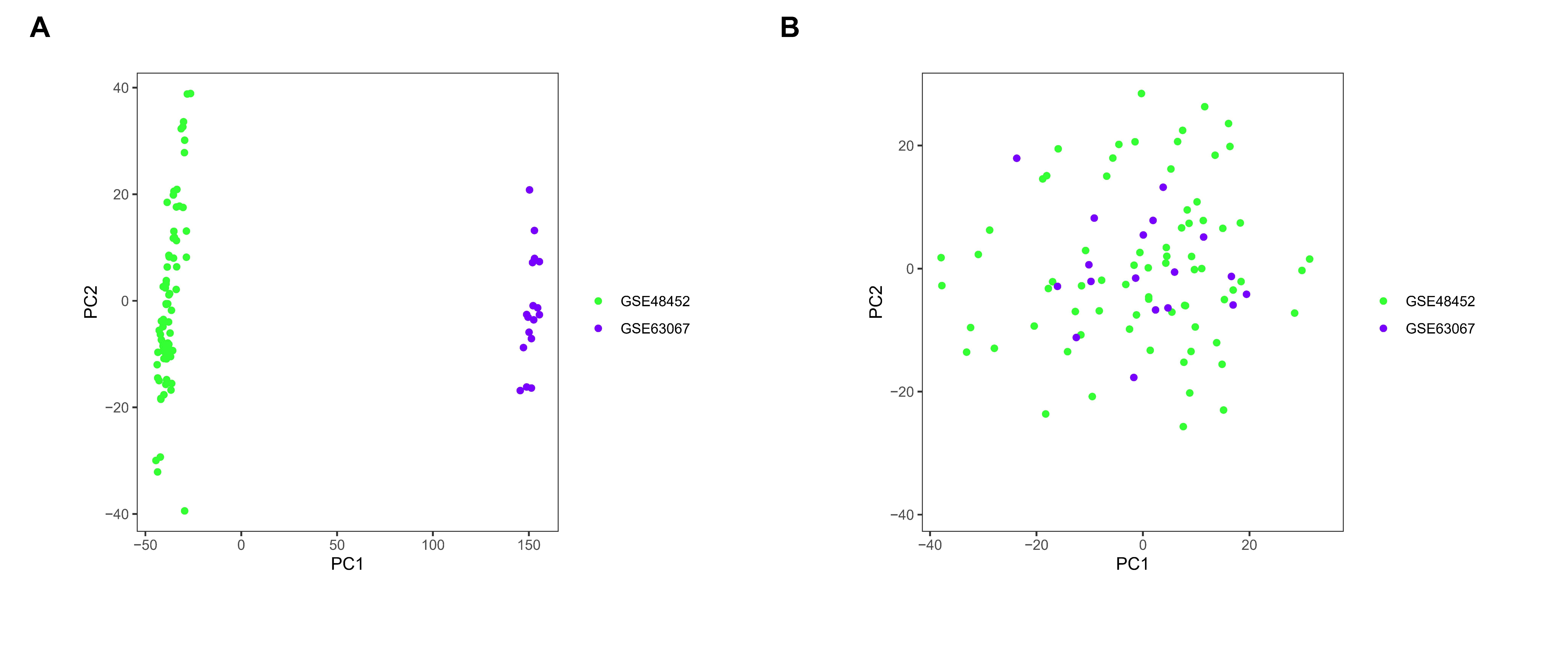

Supplement: Supplementary file 2 [file Image1.TIFF]

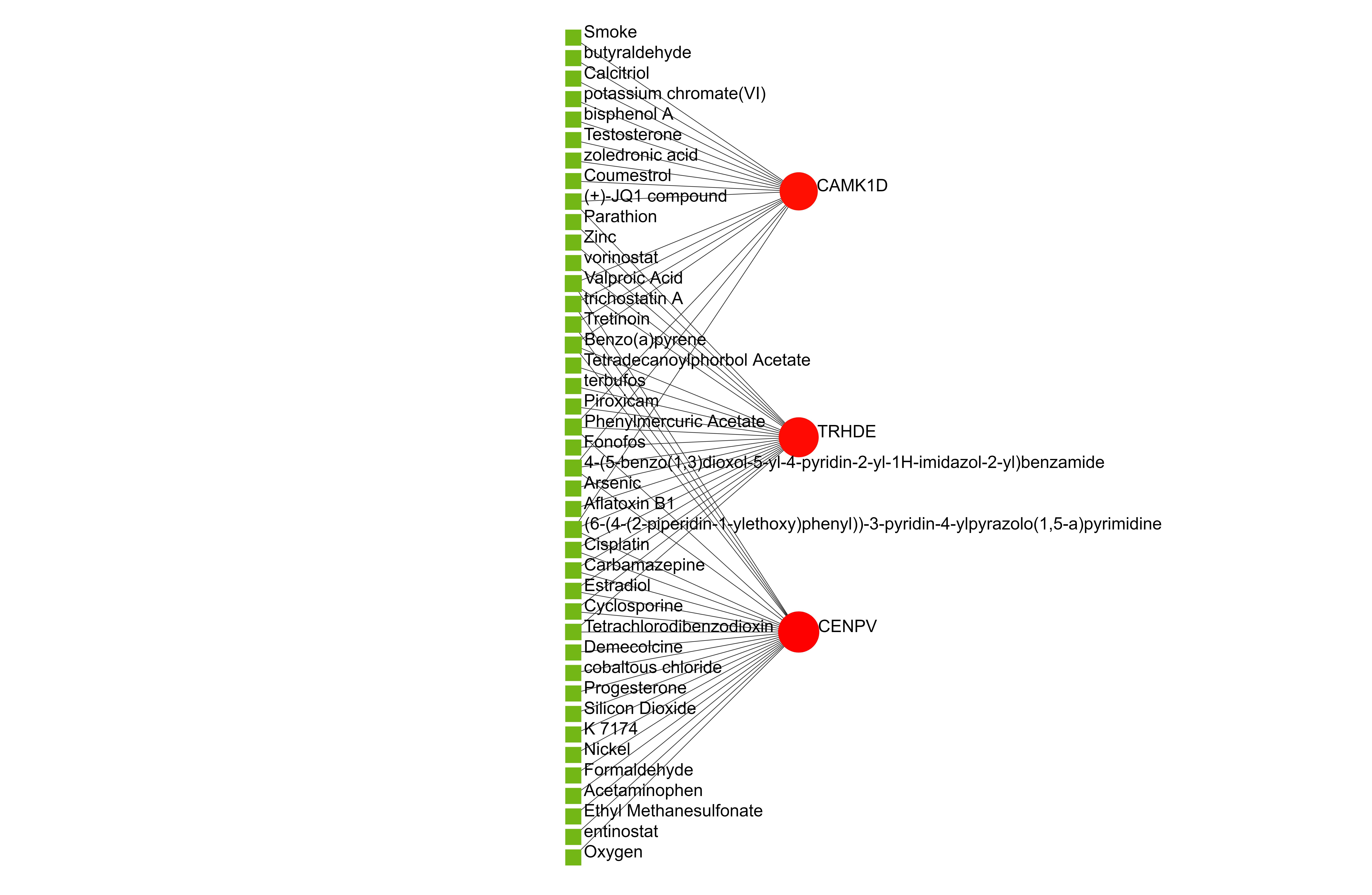

Supplement: Supplementary file 3 [file Image4.PNG]

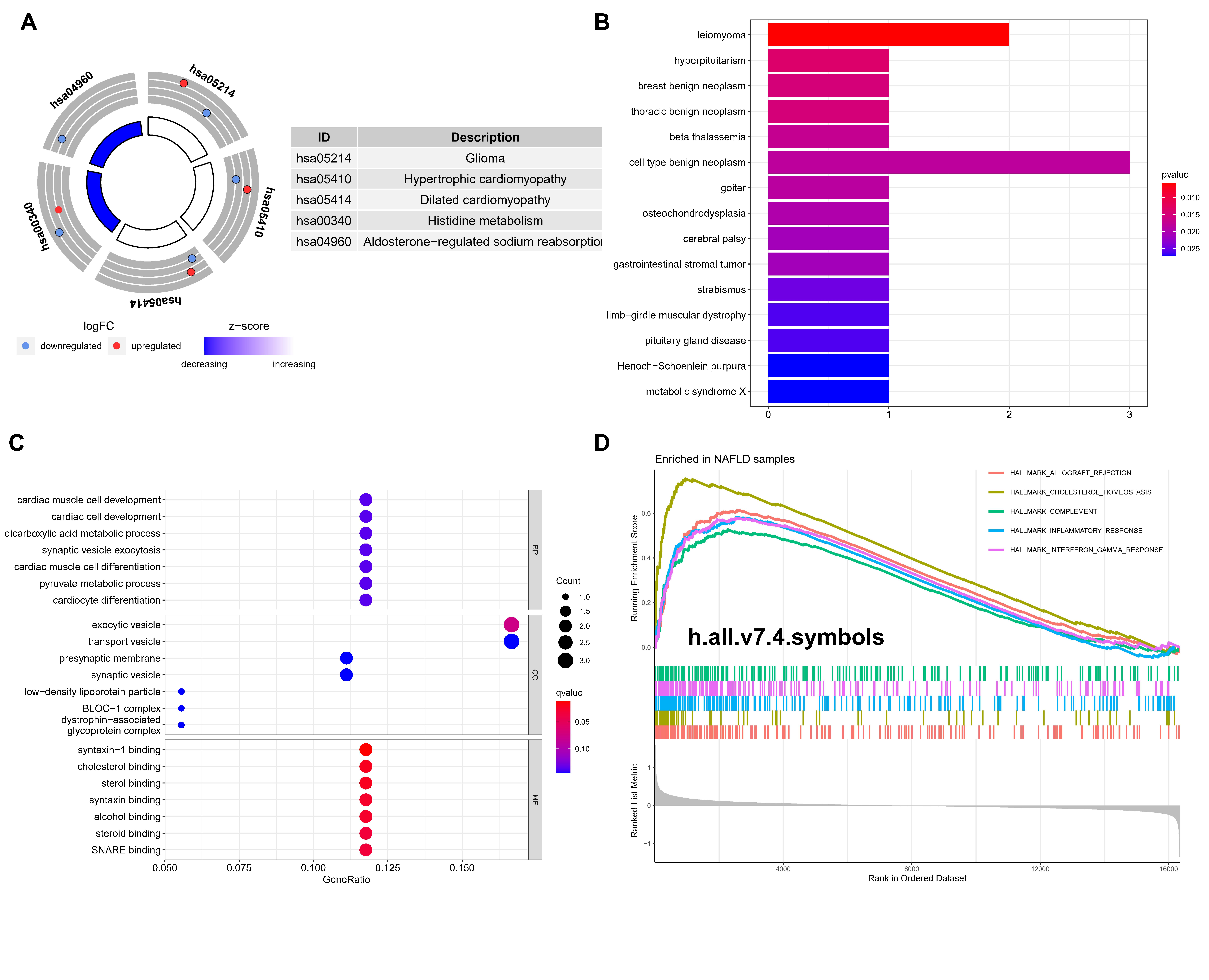

Supplement: Supplementary file 4 [file Image2.TIFF]
